# Using Leaf-Derived Materials to Stop Common Bed Bugs (*Cimex lectularius* L.) in Their Tracks

**DOI:** 10.3390/insects16080786

**Published:** 2025-07-31

**Authors:** Patrick Liu, Jorge Bustamante, Kathleen Campbell, Andrew M. Sutherland, Dong-Hwan Choe, Catherine Loudon

**Affiliations:** 1 Department of Ecology and Evolution, UC Irvine, Irvine, CA 92697, USA; liuph@uci.edu; 2 Department of Entomology, UC Riverside, Riverside, CA 92521, USAkathleen.campbell@ucr.edu (K.C.); dchoe003@ucr.edu (D.-H.C.); 3 Statewide Integrated Pest Management Program, UC Agriculture and Natural Resources, Hayward, CA 94544, USA; amsutherland@ucanr.edu

**Keywords:** horizontal, monitoring, *Phaseolus vulgaris* L.

## Abstract

The common bed bug, *Cimex lectularius* L., is a pervasive pest of humans throughout the world. Insecticide resistance, cryptic habits, and proclivity for harborage on human belongings have contributed to its global status as a difficult pest to control. Leaves from common bean plants, *Phaseolus vulgaris* L., which include numerous trichomes, have traditionally been used to capture wandering bed bugs in southeastern Europe. However, fresh leaves rapidly desiccate once removed from plants, losing their trapping ability. A leaf-derived trapping material was developed that does not desiccate as rapidly as fresh leaves and retains the potential to trap bed bugs. In this study, we tested the efficacy of the leaf-derived material in capturing bed bugs. We tested the leaf-derived material in both horizontal and vertical orientations, using fresh bean leaves as positive controls. When deployed horizontally, the leaf-derived material captured bed bugs of all life stages and both sexes (adults). Leaf-derived material was also found to capture bed bugs in a vertical orientation (only evaluated for adult male bed bugs). Because this experimental leaf-derived material was effective in both horizontal and vertical orientations and against all life stages, it may have great potential for development into bed bug monitoring or exclusion devices.

## Simple Summary

Historically, bean leaves have been used to capture wandering bed bugs. However, excised leaves dry out quickly, reducing their ability to capture bed bugs with their hooked trichomes (plant hairs). This study evaluates a new bed bug trapping material derived from bean leaves. Our analysis shows that all mobile stages of bed bugs can be caught by our experimental leaf-derived trapping material, which can be successfully deployed both horizontally and vertically.

## 1. Introduction

Bed bugs (*Cimex lectularius*, L.) are blood-feeding ectoparasites that have been recorded as human pests since ancient times [1]. They may be found wherever humans spend time sleeping or resting, such as in homes, hospitals, and hotels [2,3,4]. Bed bug infestations can cause a range of physical and mental health issues and, if unchecked, may become sources of major financial burden [5,6,7]. After many years of relative obscurity, bed bugs have resurged globally as commensal pests over the last few decades, with increasing reports of infestations since the early 2000s [8,9,10,11]. This global resurgence could be attributed to the documented evolution of resistance to commonly applied insecticides along with increases in global travel [12,13,14,15]. Use of insecticides to control bed bug populations in industrialized nations became the conventional strategy after World War II [16]. However, this convention may have resulted in bed bugs becoming resistant to many insecticides that have been commonly used in their control [17,18]. The global resurgence is now evidenced by many communities across the world producing policies and strategies to combat these pests [19,20,21,22,23,24]. To help combat the rise in bed bug infestations, physical trapping methods (e.g., pitfall traps and sticky surfaces) are commonly used to detect infestations, evaluate treatments, prevent bites (via interception), and reduce populations [11,25,26].

Another potential physical trapping method is provided by the common bean plant (*Phaseolus vulgaris*, L.). Hundreds of years ago, people in southeastern Europe used bean leaves to trap bed bugs [16]. The leaves would be placed around sleeping quarters and then disposed of once bed bugs were caught. Bed bugs are unable to escape after being trapped; the hooked trichomes on the leaf surface irreversibly pierce their legs [27]. The bed bugs eventually die on the leaf surface, probably due to desiccation or starvation [28]. However, fresh leaves are not practical as control methods as they eventually lose their ability to capture bed bugs after they are harvested, presumably due to drying. This paper evaluates a leaf-derived trapping material (patent pending, [29]) that was inspired by the action of the bean leaf.

This experimental leaf-derived trapping material could be incorporated into devices that serve as alternatives to the prevailing bed bug monitoring and exclusion methods (pitfall traps and sticky traps). To evaluate potential device limitations or constraints, we measured the distance bed bugs walked on the material before becoming trapped. Specifically, we explored any differences in the pre-capture travel distance and proportion trapped among the nymphal stages and adult sexes when walking across horizontal trapping surfaces. However, if effective, the experimental material could potentially be deployed in various orientations to meet specific monitoring or exclusion goals. For instance, bed bugs may be found on vertical surfaces such as walls and bed frames, so it would be beneficial if the experimental material was effective when deployed vertically. Trapping performance could differ by deployment orientation for two reasons: (1) locomotory patterns in insects are modified with surface angle [30,31], and (2) the direction of the forces generated between the material and the insect tarsi depends upon the surface angle [32]. Therefore, we also evaluated the effect of orientation (vertical or horizontal) on the efficacy of the leaf-derived trapping material, using fresh bean leaves (within an hour after harvest) as a comparative benchmark. To make this comparison, we considered pre-capture travel distance and the proportion of bed bugs trapped on these surfaces. We also tested whether fresh leaves could capture bed bugs once dried, to verify the anecdotal accounts of rapidly declining efficacy. Lastly, scanning electron microscopy (SEM) was used to observe the trapping mechanism of the leaf-derived trapping material.

## 2. Materials and Methods

### 2.1. Bed Bugs, Plants, and Leaf-Derived Trapping Material

#### 2.1.1. Bed Bugs

Bed bugs were fed on defibrinated rabbit blood (HemoStat Laboratories, Dixon, CA, USA) using an artificial feeding system in a laboratory at UC Riverside. Bugs were maintained at 24–26 °C and 15–30% RH, with a photoperiod of 12:12 (L:D) h. Bugs shipped to UC Irvine were temporarily held in an insect rearing room at 21 °C and 76% RH with a photoperiod of 16:8 (L:D) h without additional feeding. Bugs were fed 2–45 days before use in experiments; this range in feeding status generated no differences in observed responses (see below for statistical approaches and results).

#### 2.1.2. Bean Plants and Leaf-Derived Trapping Material

Common bean plants were raised from seeds (Johnny’s Seeds, Product 2554, https://www.johnnyseeds.com/, Winslow, ME, USA). The plants were maintained at the UC Irvine Greenhouse under natural light cycles with supplemental grow light illumination as needed by weather conditions. Individual leaves between 7 and 14 cm in length were collected by severing the petiole. Freshly collected leaves were immediately placed into sealed plastic bags to avoid desiccation and were used for both behavioral assays and chemical treatment within the hour. A subset of collected leaves was chemically treated to generate the leaf-derived trapping material (the process is patent pending, [29]). The set of leaf-derived trapping materials was then stored in sealed plastic bags at room temperature and used within six months after production.

### 2.2. The Effects of Nymphal Stages and Sex on Bed Bug Trapping

**Figure 1:**
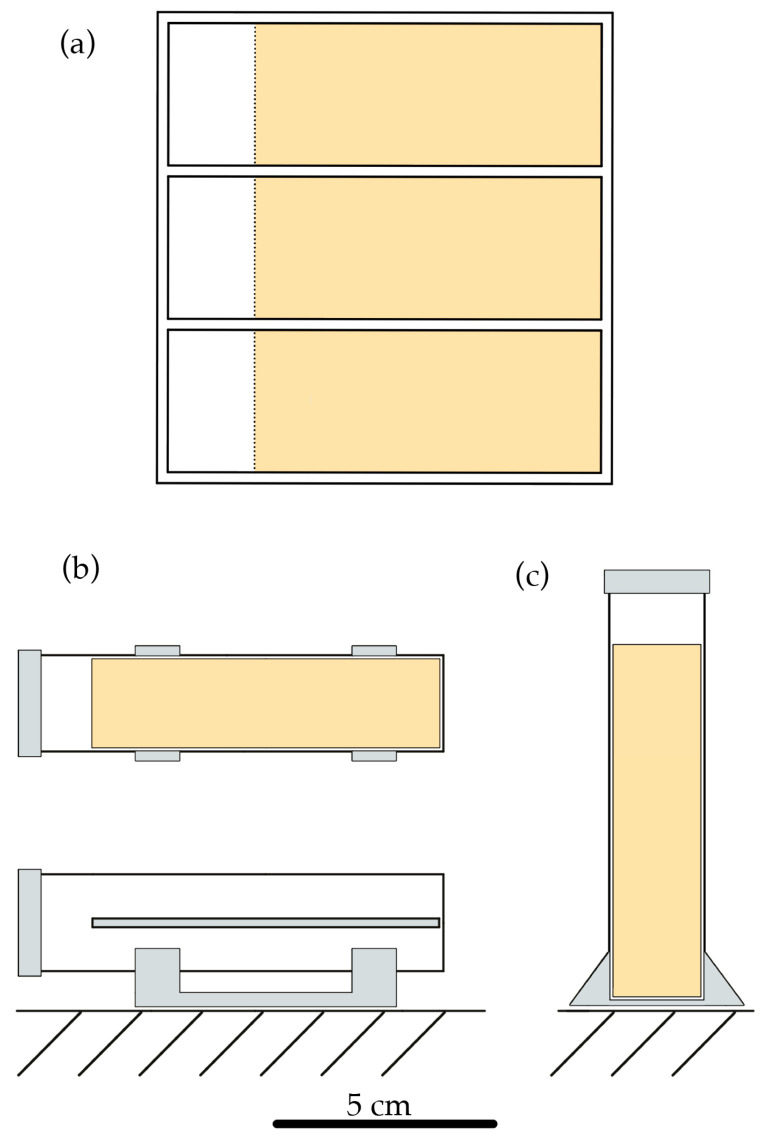
Diagram of experimental arenas: (**a**) Top view of the arena described in Section 2.2. The three compartments contained leaf-derived trapping material (yellow) with a 2 cm margin of paper on one side. Bed bugs were released on the paper and could venture onto the leaf-derived trapping material. (**b**) Top (top) and side (bottom) views of the arena described in Section 2.3 when placed in a horizontal orientation. Leaf-derived trapping material was affixed to a plastic backing and inserted into a glass culture tube. After placing the bed bugs onto the leaf-derived trapping material, the glass culture tubes were sealed and placed horizontally on a stand. (**c**) Front view of arena described in Section 2.3 when placed in a vertical orientation.

The experimental arena had three adjacent rectangular compartments with plastic walls; each compartment measured 10 cm in length × 3.2 cm width × 2.8 cm height. The bottom of each compartment was partly lined with leaf-derived trapping material; the trapping material was affixed to underlying printer paper using double-sided sticky tape (3M Scotch Double Sided tape, Saint Paul, MN, USA), with a 2 cm width of paper exposed at one end of the compartment (Figure 1a). This design enabled the observation of three bed bugs simultaneously. To prevent bed bugs from escaping, surfaces of the arena walls were coated with Fluon (Teflon PTFE DISP 30–500 mL, Fuel Cell Earth LLC, Stoneham, MA, USA). Bed bugs were released individually on the exposed paper portion of the three observation surfaces in the arena. Twenty-one bed bug individuals were used for each adult sex and the third, fourth, and fifth instar juveniles. Twelve first instar and twenty-three second instar individuals were also used.

Bed bug movements were recorded for 60 min after placement of the three bed bug arenas in a completely dark room using a near infrared camera (Basler, acA1300-69gmNIR, Exton, PA, USA) capturing 15 frames per second. The experimental arena was illuminated using infrared (Axton AT-11S, 850 nm, 150°, North Salt Lake, UT, USA). The recorded videos were then analyzed using EthoVision XT (version 11.5, Leesburg, VA, USA). The EthoVision software recorded the Cartesian points (x and y) of the bed bugs moving throughout each trial. Total distances traveled were calculated from these points; using every point (15 Hz) would lead to an inflated total distance because of the summed digitizing errors and the irrelevant side-to-side motions of the center of the body, and therefore a minimum distance of 4 mm was used to calculate successive line segments that were summed to produce the total distance traveled. Once each recording was completed, the bed bugs’ entrapment status was recorded. Bed bugs were considered trapped if they were visibly struggling in place or were still affixed to the trapping material after some stimulation (gently breathing on the insect).

### 2.3. Effects of Surface Type and Orientation (Horizontal vs. Vertical) on Bed Bug Trapping

The test surfaces (fresh leaves and leaf-derived trapping material) were cut into 2 cm × 8 cm rectangles. The rectangular surfaces were then affixed to a 3D-printed plastic backing (gray polylactic acid printer filament, Inland, Hilliard, OH, USA) with the same dimensions using double-sided sticky tape. The plastic backing with test surface was inserted into a glass culture tube (2 cm diameter, 9.5 cm length). The tube was then held either horizontally or vertically using 3D-printed plastic stands (Figure 1b,c). Individual adult male bed bugs were then placed onto the test surface through the opening in the culture tube. The glass culture tube containing the test surface and bed bug was then sealed using a 3D-printed plastic cap. Twelve bed bugs were tested for each surface and orientation combination (fresh leaves and leaf-derived trapping material; horizontal and vertical), for a total of 48 individuals. A digital camera (Panasonic GH4, Kadoma, Osaka, Japan) was set to take time-lapse videos at 1 frame per 5 s for 1 h after sealing the culture tube. A speedlight flash (Bolt, VM-160, New York, NY, USA) was used to momentarily illuminate the subject during each photograph. The experiment was conducted in an unoccupied room in complete darkness. Bed bugs were checked for entrapment status after 24 h. Bed bugs were considered trapped when they were observed (1) in the same location at the 1 h and 24 h marks, and (2) not moving from that position when gently stimulated, although displaying clear signs of life (struggling or moving their antennae). The Cartesian coordinates corresponding to the center of each insect’s body were manually recorded using Tracker software (Open Source Physics, https://opensourcephysics.github.io/tracker-website/, accessed on 1 February 2025). The distances the bed bugs moved were then calculated by summing distances between successive tracked points using Tracker. Total distances traveled were comparable using either sampling method: a minimum 4 mm length between successive points (used in Section 2.2) or a 5-s interval between successive points (used in Section 2.3) (paired *t*-test, *p* = 0.1575, *n* = 21 pairs).

### 2.4. Verification of Loss of Trapping Ability in Fresh Leaves

One subset of leaves was placed in a single layer on a plastic tray and dried for 20–24 h in a room at 21 °C and 74% RH. The ability of the dried leaves to trap bed bugs was then assessed using the same methods described in Section 2.3, considering a horizontal orientation only (12 individual bed bugs). Filming and subsequent analysis were also completed using the same methods described in Section 2.3.

To estimate the water content and water loss under these drying conditions, 10 fresh leaves were picked and immediately placed into plastic bags. The leaves were then transported to a balance (Model A-200DS, Denver Instrument Company, Denver, CO, USA) and individually weighed. Afterwards, the leaves were placed in a single layer on a plastic tray under the same drying conditions described above, and weighed again at 2, 17, and 24 h after this initial weighing. The leaves were further dried for an additional 48 h in an oven at 60 °C (Fisher Scientific Isotemp Incubator, Hampton, NH, USA) and weighed every 24 h.

### 2.5. Scanning Electron Microscopy of Bed Bugs on Leaf-Derived Trapping Material

Individual bed bugs were placed onto leaf-derived trapping material and allowed to walk until they became trapped. A piece of the material with the trapped bed bug was cut out using a scalpel and placed onto carbon tape (Product 16073, Ted Pella, Reading, CA, USA) on an SEM stub (Product 16111, Ted Pella). The specimens were then sputter coated (7 nm of Ir, Leica ACE600, Deerfield, IL, USA) and viewed using high vacuum SEM (FEI Quanta 3D FEG FIB/SEM, Waltham, MA, USA) at UC Irvine. Images of four individual bed bugs were taken.

### 2.6. Statistical Analysis

Collected data were analyzed using MATLAB R2023a (Statistics and Machine Learning Toolbox version 12.5) and SAS 9.4. Pearson’s chi-squared tests were used to compare the percent of bed bugs trapped between more than two groups (using the MATLAB crosstab function, followed by the Marascuilo procedure for post hoc analysis (using procedure described in [33]). A Cochran–Armitage test for trend was used to find additional associations between life stage (treated as an ordinal variable) and trapping percentage (using the SAS procedure PROC FREQ [34,35]). A G-test of independence was used to compare the percent of bed bugs trapped between two groups based on surface orientation, nymphal stage, or sex (using custom MATLAB script based on the procedure described in [36]). Wilcoxon rank-sum tests were used to discern differences in the pre-capture travel distance between two groups (using the MATLAB ranksum function). Kruskal–Wallis tests were used to discern differences in pre-capture travel distance between more than two groups, with post hoc analysis using Fisher’s least significant difference procedure (using the MATLAB kruskalwallis and multcompare functions). Outliers in the boxplots were determined using the interquartile range (IQR) method: values exceeding 1.5 times the IQR above the third quartile or below the first quartile were classified as outliers. All outliers were included in the non-parametric statistical tests. A Wilcoxon two-sample test was utilized to evaluate if the trapping status of a bed bug was associated with the distance it walked (using the SAS procedure PROC NPAR1WAY). The Spearman rank-order correlations procedure was used to assess the effects of days since feeding on pre-capture travel distance (using the SAS procedure PROC CORR). Logistic regression was used to assess the effect of days since feeding on entrapment status (using the SAS procedure PROC LOGISTIC). A Student’s *t*-test was used to assess differences in the mean water content of drying leaves (using the SAS procedure PROC TTEST).

## 3. Results

### 3.1. The Effects of Life Stages and Sex on Bed Bug Trapping

The leaf-derived trapping material trapped bed bugs of all life stages and both sexes. The bed bugs that became trapped were all trapped within the first hour. When checked at the end of the 24 h period, all of those bed bugs were still trapped at the same location on the material. The percent of trapped bed bugs differed significantly among nymphal instars (Pearson’s chi-squared test of independence, *χ*^2^ = 13.434, *df* = 4, *p* = 0.009, *n* = 94) (Table 1). Subsequent pairwise comparison resulted in a single significant difference between nymphal instars: the percent of fifth instar bed bugs caught was significantly different from that of second instar bugs (Marascuilo procedure, critical value = 0.317, observed difference = 0.333, *p* < 0.05). Overall, there was a significant trend detected wherein a smaller proportion of bugs were caught with each increasing instar, when treating instar as an ordinal variable (Cochran–Armitage trend test, *Z* = −3.12, *p* = 0.002, two-sided test, including all five nymphal instars). Nymphal bed bugs that became trapped travelled significantly shorter distances (median = 9.01 cm, *n* = 79) than those that were not (median = 35.5 cm, *n* = 15), suggesting that being trapped was not driven by more movement (Wilcoxon two-sample test, *W* = 955, *p* = 0.01, *n* = 94).

The observed trend of decreasing entrapment status as bed bug age increased was strengthened when adults were included as an additional life stage (Cochran–Armitage trend test, *Z* = −5.656, *p* < 0.0001, six groups including all five nymphal instars and adults as a sixth group, combining males and females). Grouping all nymphs together to compare against all adults generates a similar result, with a significantly lower percentage of adult bed bugs trapped (38.1%, 16/42; both sexes combined) than nymphal bed bugs (84.7%, 83/98; all instars combined) (*G*-test of independence, *G* = 29.608, df = 1, *p* < 0.001). Adult males were caught with greater frequency than adult females (borderline significant difference, *G*-test of independence, *G* = 3.703, df = 1, *p* = 0.054) (Table 1). Adult bed bugs that were not trapped travelled farther (median = 88.5 cm, *n* = 26) than trapped ones (median = 41.2 cm, *n* = 16) (borderline significant difference, Wilcoxon two-sample test, *W* = 269, *p* = 0.054, *n* = 42).

**Figure 2:**
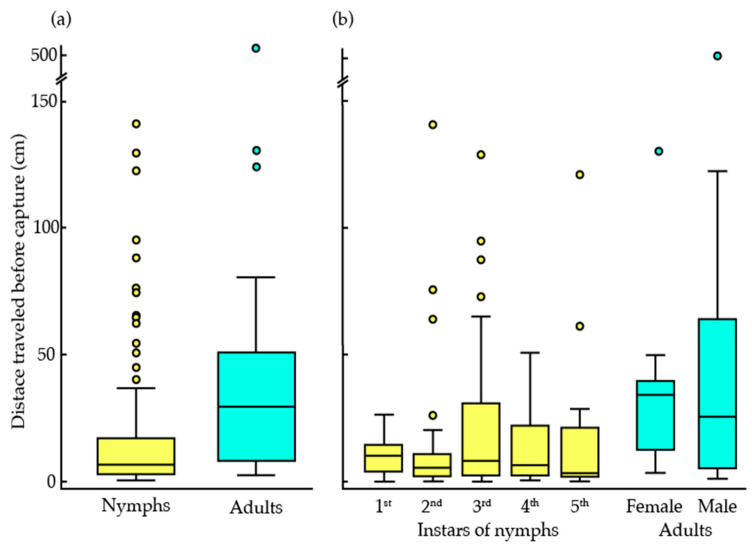
Pre-capture travel distance of bed bugs of all life stages on horizontally oriented leaf-derived trapping material: (**a**) data for immature life stages and all adults are pooled; (**b**) data are presented for each of the nymphal instars and adult sexes separately. The box represents the first through third quartiles of the data range, and the median is represented by the line inside the box. The lines extending from the box represent the minimum and maximum values of the range, excluding outliers, which are indicated by circles.

The distance that a bed bug travels on the trapping material before becoming trapped is a useful metric for evaluating the efficacy of the material for the different stages and sexes. There were no significant differences in the pre-capture travel distance between nymphal stages (Kruskal–Wallis test, *χ*^2^ = 5.24, df = 4, *p* = 0.264, *n* = 79). Adults were trapped at significantly longer travel distances (median = 41.2 cm, *n* = 16) than nymphs (median = 9.0 cm, *n* = 79) (Wilcoxon rank-sum test, *W* = 1083, *p* = 0.002, *n* = 95) (Figure 2). Pre-capture travel distances were not significantly different between adult sexes (Wilcoxon rank-sum test, *W* = 35, *p* = 0.428, *n* = 16) (Figure 2).

### 3.2. Effects of Surface Type and Orientation (Horizontal vs. Vertical) on Trapping Bed Bugs

Whether the leaf-derived trapping material can capture bed bugs in orientations other than horizontal is important to evaluate because the ability to deploy this material in different orientations would greatly expand its utility in pest management. Having established that the leaf-derived material can capture bed bugs of all life stages and both sexes in a horizontal configuration (Section 3.1), we focused further analysis on orientation using adult male bed bugs and including fresh bean leaves as benchmark comparisons. Analyses in this section do not include data already analyzed in Section 3.1. More than half of adult male bed bugs were trapped within an hour by both fresh leaves and leaf-derived trapping material used in either orientation (Table 2) (bugs were still trapped in the same location when checked again an additional 23 h later). The percentage of trapped bugs did not significantly differ between the four surface × orientation combinations (fresh leaves and leaf-derived trapping material, horizontal and vertical) (Pearson’s chi-squared test of independence, *χ*^2^ = 2.8484, df = 3, *p* = 0.4156, *n* = 48). There was no significant effect of trapping surface or orientation on trapping percentage (G-test of independence, surface: *G* = 0.9560, df = 1, *p* = 0.3282; orientation: *G* = 0.9560, df = 1, *p* = 0.3282). Feeding status was not associated with being trapped (logistic regression, trapped status as binary outcome and days since fed as a covariate, *χ*^2^ = 0.1224, df = 1, *p* = 0.7265, *n* = 48).

**Figure 3:**
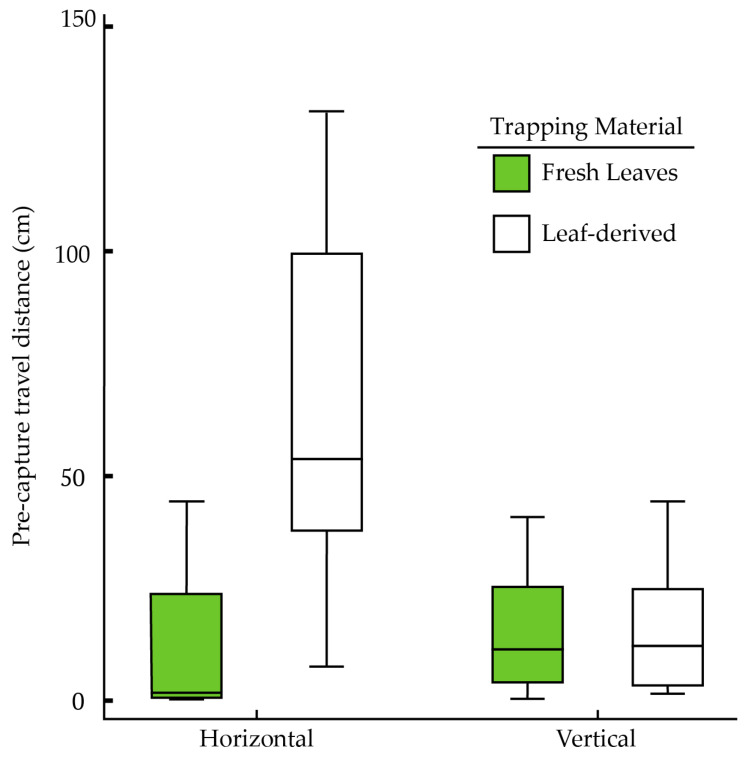
Pre-capture travel distance of adult male bed bugs on two different trapping surfaces in two orientations (fresh leaves vs. leaf-derived trapping material, horizontal vs. vertical) (*n* = 11 for fresh leaves horizontal, *n* = 8 for other treatments). The figure shows boxplots comparing the distance bed bugs walked before capture on either fresh leaves (green) or leaf-derived trapping material (white) in two different orientations.

There was a significant difference in pre-capture travel distances when comparing all the surface × orientation combinations (Figure 3) (Kruskal–Wallis, χ^2^ = 13.32, df = 3, *p* = 0.004, *n* = 48). Post hoc analysis revealed that the pre-capture travel distances were significantly longer for the horizontally oriented leaf-derived trapping material than all the other surface × orientation combinations (Fisher’s least significant difference procedure, *p* < 0.05). The pre-capture travel distance was significantly longer on the leaf-derived trapping material than on fresh leaves (combining both orientations, Wilcoxon rank-sum test, *W* = 240, *p* = 0.010, *n* = 48). There was no significant difference in the pre-capture travel distance between horizontal and vertical orientations (combining both surfaces, Wilcoxon rank-sum test, *W* = 356, *p* = 0.425, *n* = 48) (Figure 3). Days since being fed did not significantly affect distance travelled (Spearman rank-order correlation, *r* = −0.142, *p* = 0.33, *n* = 48).

### 3.3. Verification of Loss of Trapping Ability in Fresh Leaves

To verify the loss of trapping ability, we dried fresh leaves for one day (24 h) at room conditions, before placing the bed bugs on the leaves. Consistent with earlier observations, none of the bed bugs placed on the dried leaves were trapped within one hour (0/12 trapped, average distance walked 55 cm, range 3.1 cm–173.2 cm). The bed bugs were checked again after another 23 h, and still none were trapped. For comparison, using this average distance of 55 cm, 92% of the bed bugs walking on fresh leaves (11/12) and 42% walking on the leaf-derived trapping material (5/12) were caught within 55 cm.

The fresh leaves lost an average of 52% of their water content in 24 h under room conditions (range 45–62%, *n* = 10; one-sided paired *t*-test, loss significantly > 0, *p* < 0.001, *n* = 10). The total water content of the fresh leaves (immediately after being picked) was 86% of their total weight (range 83–88%, *n* = 10), as estimated from the fresh weight—dried weight (dried at 60 °C for 48 h). This corresponded to an average of 6.1 g water/g dry mass in the fresh leaves (range 5.0–7.7, *n* = 10), which is a typical value for fresh leaves [37]. The leaves were no longer losing weight after the first 24 h at 60 °C (one-sided paired *t*-test, *p* = 0.9965, *n* = 10).

### 3.4. Scanning Electron Microscopy of Bed Bugs on Leaf-Derived Trapping Material

**Figure 4:**
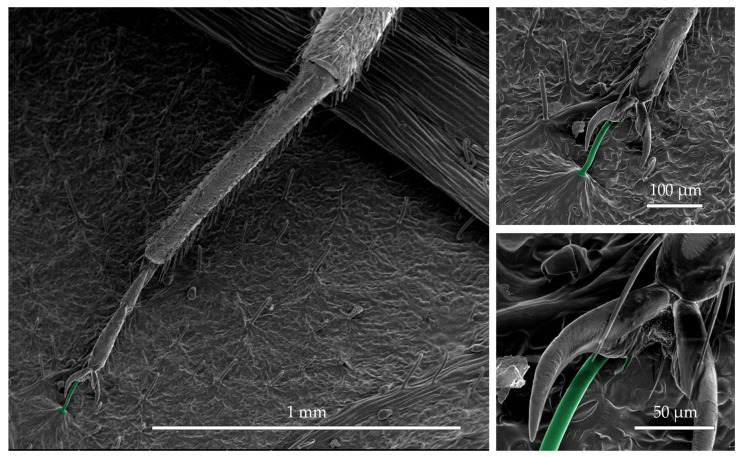
Scanning electron micrographs of a hooked trichome on leaf-derived trapping material piercing the undersurface of the pretarsus of a bed bug midleg. The hooked trichome piercing the pretarsus is falsely colored in green. The micrographs on the right are higher magnification views of the same leg shown on the left.

The hooked trichomes on the surface of the leaf-derived trapping material were observed to pierce the legs of bed bugs in a similar manner documented for trichomes on fresh leaves [27]. Most of the piercings we observed (4/7) were at the undersurface of the bed bug’s pretarsal claws (Figure 4). Piercings were also observed at the joints between subsegments on the tarsi (3/7). The four bugs used for verification of piercing all showed evidence of at least one leg being pierced.

## 4. Discussion

The global resurgence of bed bugs and their increasing insecticide resistance necessitates the development of novel non-chemical methods to aid in their control. Although bean leaves have historically been used to capture bed bugs, their effectiveness is lost over time after being picked. The leaf-derived trapping material evaluated here addresses this limitation; the material continues to capture bed bugs long after fresh leaves have ceased to work. In this study, we evaluated the effectiveness of the leaf-derived trapping material in capturing the different nymphal stages and both sexes of adult bed bugs. We then compared the effectiveness of fresh bean leaves and the leaf-derived trapping material in two different orientations. We also verified that fresh leaves lose their trapping ability after desiccation. Finally, SEM analysis of the trapping mechanics of the leaf-derived trapping material was conducted, revealing the physical entrapment mechanism.

To assess the trapping efficiency of the leaf-derived trapping material, we measured the percentage of bed bugs that were captured during one hour of observation. The leaf-derived trapping material captured bed bugs of all life stages and sexes. However, there was a trend of decreasing percentage of nymphs caught with advancing nymphal stage. This trend continued with the adults, as they exhibited a markedly lower incidence of capture compared with the nymphal stages, with females having the lowest (24%). Another measure of efficiency is the distance bed bugs travel over the material before becoming trapped. Although this metric is not routinely reported for trapping surfaces (for the exception, see [27]), it provides a quantitative basis for comparison of alternative methods. Adult bed bugs of both sexes were caught after significantly longer distances than any of the nymphal stages (approximately four times greater).

Why are nymphs captured more quickly and frequently than adults? The mechanism of capture by the leaf-derived trapping material is the same as that of fresh bean leaves; their trichome hooks pierce the cuticle of the bed bugs’ tarsi, permanently entrapping them (Figure 4) [27,28]. Adult bed bugs are larger and presumably stronger than nymphs, and therefore could potentially pull themselves off the hooks, whereas nymphs may be unable to do so. The smaller size of the nymphs also means that they take shorter and more frequent steps than adults for any given distance. The greater number of steps would give more chances for the trichome hooks of the leaf-derived trapping material to engage the bed bug’s tarsi for a given distance of travel.

Experimental materials were evaluated in both horizontal and vertical orientations to simulate different potential application methods (e.g., on bed legs vs. a flat trap on the floor under a bed). Both fresh leaves and the leaf-derived trapping material captured bed bugs in both orientations, although there were some differences in trapping performance. Most bed bugs (>67%) placed onto either surface at both orientations were captured within 24 h. In the horizontal orientation, fresh leaves were more effective than the leaf-derived trapping material; bed bugs traveled approximately thirty times farther on the leaf-derived trapping material before capture. However, the leaf-derived trapping material was just as effective as fresh leaves in the vertical orientation (no significant differences in either percent trapped or distance traveled before trapping). This result suggests that the performance of leaf-derived trapping material is orientation-dependent, being more effective in a vertical orientation. Fresh leaves did not exhibit a similar orientation effect (Figure 3). Differences in capture distances for the leaf-derived trapping material may be explained by insects altering their locomotion in response to walking on different surface textures and orientations. Bed bugs rely on pretarsal claws to grip surfaces [38,39,40], which are limited by the smoothness of a surface [41,42]. Pretarsal claws would have to interlock with the surface, unlike tarsal pads, which contain specialized structures (e.g., arolia and pulvilli) that conform to the shape of the surface [43,44]. The leaf-derived trapping material is noticeably thinner and more flexible than fresh leaves, changes potentially caused by the chemical treatment altering its cellulose fibers [45]. These changes could also include alterations to the surface texture, making the surface more difficult for the bed bug to grip, possibly leading to more slipping, especially along a vertically oriented surface. With respect to orientation, the changes in the direction of gravitational forces relative to the foot contact with the surface require some insects to alter their walking speed and step duration to maintain stability and purchase [1,46]. A vertical orientation is likely to cause bed bugs to take shorter and more frequent steps. This, in turn, would provide more opportunities for trichome hooks to engage the bed bug’s tarsi. Detailed locomotory differences are yet to be quantified for bed bugs walking on these surfaces.

Many monitoring and interceptor devices exploit the weak climbing ability of bed bugs on smooth surfaces (e.g., pitfall traps). However, recently molted bed bugs exhibit an enhanced ability to climb smooth surfaces [47], potentially enabling them to escape these devices and avoid detection. Similarly, the tropical bed bug (*Cimex hemipterus*), which has also seen a resurgence, is able to climb smooth surfaces and escape pitfall traps [13,48,49,50,51]. Unlike these methods, the leaf-derived trapping material does not rely on smooth surfaces and therefore could possibly be used in the management of different bed bug species, including recently molted individuals. However, it is yet to be tested against *C. hemipterus* or recently molted individuals of either bed bug species.

Fresh leaves were found to lose the ability to trap bed bugs within 24 h after being picked under room conditions. The loss of this ability is most likely caused by the effects of drying. Most of the water content of the leaves had evaporated after 24 h (21 °C, 74% RH). In contrast, the leaf-derived trapping material is stable after its processing, can be stored in containers indefinitely under room conditions, and continues to capture bed bugs weeks to months after being produced.

Additional work is needed to fully evaluate how the leaf-derived trapping material could be deployed in pest control efforts. Traps using this material have been shown to be more effective than selected pitfall and sticky traps in laboratory evaluations [52]. Using insights from the current study, we can determine the size of the leaf-derived trapping material needed to achieve a target trapping probability. For example, from the observed distances traveled by bed bugs before capture, a 10 cm wide strip of the trapping material would be expected to capture 52% of immature bed bugs and 25% of adult bed bugs that walked across the surface once. Future work will focus on evaluating the potential utility of devices incorporating the leaf-derived trapping material in more realistic field settings and identifying conditions under which they may outperform commercially available products.

## Figures and Tables

**Table 1:** Percent of bed bugs trapped on leaf-derived trapping material at different life stages and sexes.

Life Stage	Bugs Trapped (% Trapped, Trapped/Total Number)
All Bugs	68% (95/140)
1st Instar	92% (11/12)
2nd Instar	100% (23/23)
3rd Instar	95% (20/21)
4th Instar	71% (15/21)
5th Instar	67% (14/21)
Adult (females)	24% (5/21)
Adult (males)	52% (11/21)

**Table 2:** The percent of adult male bed bugs trapped on fresh leaves or leaf-derived trapping material at different orientations.

Orientation	Fresh Leaves (% Trapped/Total Number)	Leaf-Derived Trapping Material (% Trapped/Total Number)
Horizontal	92% (11/12)	67% (8/12)
Vertical	67% (8/12)	67% (8/12)

## Data Availability

The raw data supporting the conclusions of this article will be made available by the authors on request.
